# Functional Recovery in a Child With Adrenoleukodystrophy Following Neuroregenerative Effects of Radio Electric Asymmetric Conveyer (REAC) Neuro-Regenerative (RGN-N) Treatment: A Detailed Case Report

**DOI:** 10.7759/cureus.74283

**Published:** 2024-11-23

**Authors:** Salvatore Rinaldi, Arianna Rinaldi, Vania Fontani

**Affiliations:** 1 Research Department, Rinaldi Fontani Foundation, Florence, ITA; 2 Department of Regenerative Medicine, Rinaldi Fontani Institute, Florence, ITA; 3 Department of Adaptive Neuro Psycho Physio Pathology and Neuro Psycho Physical Optimization, Rinaldi Fontani Institute, Florence, ITA

**Keywords:** hematopoietic stem cell transplantation (hsct), loes score, neurobiological modulation, neuro regenerative treatment, reac neuroregenerative therapy, x- linked adrenoleukodystrophy

## Abstract

This case report describes the treatment of a 9-year-old boy with advanced adrenoleukodystrophy (ALD) who received radioelectric asymmetric conveyer (REAC) neuro-regenerative (RGN-N) therapy after hematopoietic stem cell transplantation (HSCT) failed to produce therapeutic benefits. ALD is a devastating neurodegenerative disorder for which limited treatment options exist, and interventions are often ineffective in advanced cases. Post-transplant, the patient’s symptoms worsened until REAC RGN-N therapy was introduced. After treatment, notable improvements were observed in motor function, swallowing, spasticity, and overall quality of life. These results suggest that REAC RGN-N treatment may be an effective intervention to slow neurodegenerative progression and support recovery in ALD cases unresponsive to HSCT.

## Introduction

Adrenoleukodystrophy (ALD) is a rare, inherited disorder [1] caused by mutations in the ABCD1 gene, leading to the accumulation of very long-chain fatty acids (VLCFAs) in the white matter of the brain and adrenal cortex [2]. Childhood cerebral ALD, the most severe form, typically presents between ages four and 10, with early signs such as behavioral changes, motor difficulties, and learning disabilities. The disease progresses rapidly, resulting in spasticity, cognitive impairment, and loss of vision and hearing. Without intervention, ALD leads to death in many cases.

Hematopoietic stem cell transplantation (HSCT) remains the only potentially curative treatment in early-stage ALD, aiming to slow the progression of demyelination [3]. However, HSCT must be administered before significant neurological damage has occurred to be effective [4]. Even with a successful transplant, there is no guarantee of halting disease progression. In cases where the disease is advanced, or HSCT is ineffective, management is typically palliative, addressing symptoms to improve quality of life.

In this case, a boy diagnosed with ALD at the age of six experienced worsening disease despite undergoing HSCT. The disease progression prompted the exploration of alternative therapeutic approaches, leading to the introduction of radioelectric asymmetric conveyer (REAC) neuro regenerative (RGN-N) treatment [5]. This non-invasive approach uses radioelectric fields asymmetrically conveyed to modulate endogenous cellular bioelectrical activity [6] and stimulate neurofunctional reorganization [7-9]. This report details the functional recovery observed after the treatments.

## Case presentation

Patient information

The patient, born on December 2014, was diagnosed with ALD at the age of six. His family history revealed non-consanguineous parents and a maternal uncle who died at age four from ALD complications. Before diagnosis, the child showed signs such as hyperactivity, excessive drooling, and a right-sided limp linked to hemiparesis, raising concerns about potential neurological abnormalities. Brain imaging (MRI) revealed extensive cerebral demyelination with a Loes score of 10, indicating the early yet significant spread of the disease [10]. Further diagnostic testing revealed adrenal insufficiency, with cortisol levels at 3 mcg/dL and Adrenocorticotropic hormone (ACTH) at 1250 pg/mL.

In April 2021, a hematopoietic stem cell transplantation (HSCT) was performed, with the father serving as a 50% compatible donor. While the procedure succeeded technically, it failed to halt disease progression. The Loes score continued to increase, reaching 22 within 18 months post-transplant, with worsening clinical symptoms [10]. Over time, the patient developed tetraparesis, near-total vision loss, severe dysphagia, aphasia, and frequent episodes of aspiration pneumonia due to an impaired swallowing reflex. Additional complications included involuntary biting, intestinal dysbiosis, and severe spasticity coupled with generalized dystonia and myoclonus, which led to considerable discomfort and diminished quality of life. Clinical course

The ineffective transplant led to a focus on symptom management, yet conventional pharmacological options offered limited relief. Initially, the patient received baclofen for muscle spasticity, clobazam for seizure control, and hydrocortisone for adrenal support. However, symptoms persisted, necessitating adjustments in therapy, which included the introduction of cannabidiol and prednisolone. A gastrostomy was performed in March 2023 to address severe dysphagia and malnutrition, although this intervention provided only marginal improvement in quality of life.

Imaging findings

The patient underwent a follow-up cranial MRI on August 2023, which revealed further progression of the disease. Comparative analysis with the January 2023 MRI indicated a slight increase in signal alterations affecting the white matter of the frontal lobes, extending to the genu and body of the corpus callosum. This progression was evident as hyperintense areas on T2/FLAIR sequences and hypointense areas on T1 sequences, without gadolinium enhancement, though peripheral diffusion restriction was mildly observed.

**Figure 1:**
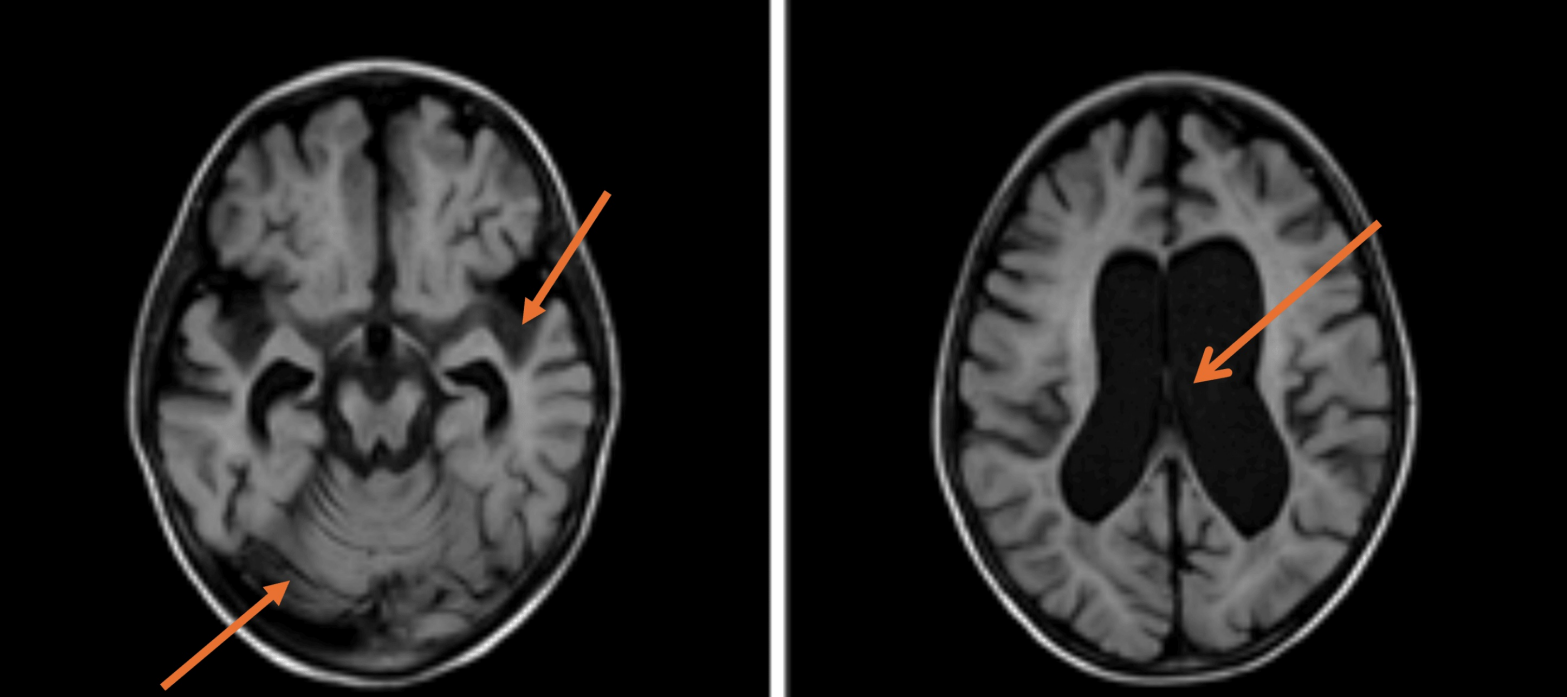
August 2023 cranial MRI The MRI reveals progression in adrenoleukodystrophy, with increased white matter signal alterations in the frontal lobes, corpus callosum, and parieto-occipital regions. Diffuse brain atrophy is indicated by ventricular enlargement, (orange arrows) widened cortical sulci (orange arrows). Loes score remains at 22

The parieto-occipital periventricular and subcortical regions continued to show similar involvement, with signal alterations also extending to the cerebellar hemispheres bilaterally. The corpus callosum’s splenium, medial and lateral geniculate bodies, optic radiations, Meyer’s loop, and fornix columns remained affected, along with the corticospinal tract from the internal capsule through the brainstem to the cervico-medullary junction, and into the spinal cord. Additionally, there was marked supratentorial ventricular enlargement, increased cortical sulci, and intracranial fissures, indicative of diffuse brain atrophy. The corpus callosum appeared thinned, particularly in the trunk, isthmus, and splenium, consistent with advanced cerebral atrophy. The Loes score, a measure of disease severity, remained elevated at approximately 22, further emphasizing disease progression [10]. Importantly, no gadolinium enhancement was detected, ruling out active neuroinflammation (Figure 1).

Therapeutic intervention

With the patient's condition continuing to decline, alternative therapies were considered. In September 2023, the REAC RGN-N treatment was initiated [5]. Each treatment cycle was conducted over 72 hours, broken into daily six-hour sessions with the Asymmetric Conveyer Probe positioned along the spine. A second treatment cycle was administered in February 2024, approximately five months after the initial cycle, allowing time for physiological adaptation. Supportive therapies, including physical, visual, and speech therapy, were continued throughout this period to aid motor, sensory, and swallowing functions, potentially enhancing the effects of REAC RGN-N.

REAC RGN-N treatment employs low-intensity radioelectric fields conveyed asymmetrically to target and modulate endogenous cellular bioelectrical activity [11,12]. This approach is designed to correct bioelectrical imbalances within cells that are often implicated in neurodegenerative diseases [13]. By stimulating neurofunctional reorganization, the treatment aims to support neurological recovery and reduce inflammation that may contribute to symptoms. Additionally, the disappearance of intestinal dysbiosis was noted, contributing to an overall improvement in the patient's gastrointestinal health.

Outcomes

Following the first cycle of REAC RGN-N treatment in September 2023 and the second cycle in February 2024, the patient demonstrated significant clinical improvements across multiple functional domains six months after the second cycle. Weekly supportive therapies, including motor physiotherapy, visual therapy, and speech therapy focused on auditory and swallowing functions, continued throughout this period and may have enhanced the treatment effects.

Swallowing function for liquids showed marked improvement, as reflected in advancement on the Functional Oral Intake Scale (FOIS), suggesting a transition from non-oral to partial oral feeding capabilities. In terms of postural control, the patient achieved the ability to maintain an upright position with support, aided by foot orthoses, indicating improvement on the Gross Motor Function Classification System (GMFCS). Additionally, neck control improved, supporting better head positioning and stability. Voluntary motor function in both upper and lower limbs also progressed as the patient regained control over movements in these areas. This was accompanied by a reduction in muscle spasticity, which improved from Grade IV to Grade II on the Modified Ashworth Scale (MAS), denoting a significant decrease in muscle stiffness and greater ease in passive movements.

Sensory responsiveness improved, with enhanced auditory localization and visual fixation capabilities, including the ability to track visual stimuli such as images and colored screens. Observational ratings of auditory and visual attention suggested heightened engagement with external stimuli, indicative of improved sensory integration. Endocrine function also stabilized, with cortisol levels normalizing at 11.20 mcg/dL and adrenocorticotropic hormone (ACTH) level at 125 pg/mL by August 2024, allowing for the discontinuation of daily prednisolone use. Additional medication adjustments were made, with the cessation of cannabidiol and clobazam, and a simplified regimen of baclofen 10 mg daily, administered as half a tablet every eight hours, was continued to manage residual spasticity. These outcomes suggest that the REAC RGN-N treatment, combined with supportive therapies, contributed to functional gains and symptomatic relief in this patient, underscoring its potential therapeutic value in addressing complex neurodegenerative symptoms.

## Discussion

This case highlights the potential therapeutic impact of REAC RGN-N treatment for managing ALD symptoms in advanced stages. Although hematopoietic stem cell transplantation (HSCT) was performed without procedural complications, its failure to halt disease progression underscores the urgent need for innovative therapeutic options. REAC RGN-N treatment, administered in 72-hour cycles with five-month intervals, demonstrated considerable improvements in motor control, sensory engagement, and spasticity reduction.

Specific quantitative comparisons support the novelty of these findings. For instance, spasticity reduction from Grade IV to Grade II on the Modified Ashworth Scale (MAS) aligns with prior reports of REAC technology’s efficacy in managing motor dysfunctions [13-15]. However, the pronounced sensory responsiveness and postural control observed in this case exceed outcomes reported in similar neurodegenerative conditions [15,16]. This underscores the possibility of unique mechanisms of action when applying REAC RGN-N in advanced ALD, a hypothesis requiring further investigation.

The stabilization of endocrine function, reflected in normalized cortisol and ACTH levels, is particularly noteworthy. While prior studies on REAC technology primarily focus on neurological improvements, these endocrine effects highlight broader systemic impacts. This finding, though novel, necessitates exploration of the interplay between neuroendocrine regulation and bioelectrical activity [17,18]. Interestingly, the absence of neuroinflammatory markers in this case contrasts with studies that emphasize REAC’s anti-inflammatory effects [15,16]. This suggests that the treatment’s efficacy in this context might be linked to alternative pathways, such as modulation of oxidative stress or correction of metabolic imbalances associated with advanced neurodegeneration. The absence of active inflammation may also indicate the advanced disease stage, where inflammation is no longer a primary driver of pathology. Importantly, the spacing of treatment cycles likely played a role in reinforcing bioelectrical stability, enhancing the long-term effects. Cellular adaptation facilitated by interval-based administration mirrors findings in regenerative medicine, where periodic treatments are associated with improved physiological integration and stability [11,13].

The concurrent use of supportive therapies, including physical, speech, and visual therapies, was maintained as part of the patient’s care plan. However, given the progressive and irreversible nature of functional loss in ALD, as documented in existing literature, these therapies are unlikely to account for the observed recovery in motor, sensory, and auditory functions. To date, there is no evidence suggesting that such therapies alone can restore these abilities once lost, further supporting the potential role of REAC RGN-N therapy in achieving these outcomes. Despite the promising outcomes, certain limitations must be acknowledged. This report focuses on a single case, which inherently limits the generalizability of the findings. However, it is important to emphasize that adrenoleukodystrophy (ALD) is a rare neurodegenerative disease, making large-scale studies inherently challenging. Moreover, to date, no treatment has proven effective in improving symptoms or slowing the clinical course of advanced ALD, further highlighting the significance of these observed improvements and the need for continued exploration of innovative therapeutic approaches like REAC RGN-N therapy.

This case illustrates that bioelectrical modulation with REAC RGN-N therapy represents a paradigm shift in addressing neurodegenerative diseases, transitioning from symptomatic management to targeting core cellular dysfunctions. The improvements in motor control, sensory responsiveness, and quality of life, despite advanced disease indicators like a high Loes score and extensive cerebral atrophy, are noteworthy. Future studies should explore the broader application of this therapy in neurodegenerative and neuroendocrine conditions to validate its efficacy and potential as a therapeutic tool.

## Conclusions

This case underscores the significant improvements in motor and sensory functions, spasticity, and overall quality of life observed following REAC RGN-N treatment in a patient with advanced ALD. The progressive recovery in motor control, sensory responsiveness, and reduction in spasticity highlights the potential of bioelectrical modulation as a therapeutic tool for neurodegenerative conditions. While further studies are needed to validate these findings and explore REAC RGN-N treatment's broader applications, this report adds to the growing body of evidence supporting the use of bioelectrical therapies in complex neurodegenerative conditions, potentially offering hope for patients in whom conventional therapies fail to halt disease progression.
